# Comparison of pretrained transformer-based models for influenza and COVID-19 detection using social media text data in Saskatchewan, Canada

**DOI:** 10.3389/fdgth.2023.1203874

**Published:** 2023-06-28

**Authors:** Yuan Tian, Wenjing Zhang, Lujie Duan, Wade McDonald, Nathaniel Osgood

**Affiliations:** Department of Computer Science, University of Saskatchewan, Saskatoon, SK, Canada

**Keywords:** influenza, COVID-19, social media, transformer-based language models, digital surveillance

## Abstract

**Background:**

The use of social media data provides an opportunity to complement traditional influenza and COVID-19 surveillance methods for the detection and control of outbreaks and informing public health interventions.

**Objective:**

The first aim of this study is to investigate the degree to which Twitter users disclose health experiences related to influenza and COVID-19 that could be indicative of recent plausible influenza cases or symptomatic COVID-19 infections. Second, we seek to use the Twitter datasets to train and evaluate the classification performance of Bidirectional Encoder Representations from Transformers (BERT) and variant language models in the context of influenza and COVID-19 infection detection.

**Methods:**

We constructed two Twitter datasets using a keyword-based filtering approach on English-language tweets collected from December 2016 to December 2022 in Saskatchewan, Canada. The influenza-related dataset comprised tweets filtered with influenza-related keywords from December 13, 2016, to March 17, 2018, while the COVID-19 dataset comprised tweets filtered with COVID-19 symptom-related keywords from January 1, 2020, to June 22, 2021. The Twitter datasets were cleaned, and each tweet was annotated by at least two annotators as to whether it suggested recent plausible influenza cases or symptomatic COVID-19 cases. We then assessed the classification performance of pre-trained transformer-based language models, including BERT-base, BERT-large, RoBERTa-base, RoBERT-large, BERTweet-base, BERTweet-covid-base, BERTweet-large, and COVID-Twitter-BERT (CT-BERT) models, on each dataset. To address the notable class imbalance, we experimented with both oversampling and undersampling methods.

**Results:**

The influenza dataset had 1129 out of 6444 (17.5%) tweets annotated as suggesting recent plausible influenza cases. The COVID-19 dataset had 924 out of 11939 (7.7%) tweets annotated as inferring recent plausible COVID-19 cases. When compared against other language models on the COVID-19 dataset, CT-BERT performed the best, supporting the highest scores for recall (94.8%), F1(94.4%), and accuracy (94.6%). For the influenza dataset, BERTweet models exhibited better performance. Our results also showed that applying data balancing techniques such as oversampling or undersampling method did not lead to improved model performance.

**Conclusions:**

Utilizing domain-specific language models for monitoring users’ health experiences related to influenza and COVID-19 on social media shows improved classification performance and has the potential to supplement real-time disease surveillance.

## 1. Introduction

Influenza and coronavirus disease (COVID-19) are highly contagious respiratory diseases imposing substantial mortality, morbidity and health system burden. In developed countries, influenza and COVID-19 surveillance systems act as a cornerstone of disease outbreak monitoring, and are essential to guide appropriate public health interventions. In Canada, traditional disease surveillance systems for influenza, influenza-like illnesses (ILIs) and COVID-19 are mostly passive and have limitations in detecting outbreaks in real-time, as they mostly rely on laboratory reports of positive cases, sentinel practitioner and syndromic surveillance programs, and provincial/territorial assessment of the geographic spread of influenza and COVID-19 (1,2). The rise of the web and social media have raised new prospects for expanding traditional surveillance of influenza and COVID-19, especially through the use of Internet- or social media-based streams as a complementary data source for real-time digital disease surveillance (3). In addition, recent advances in artificial intelligence through machine learning techniques and natural language processing (NLP) have contributed new means of extraction of key information from social media text data on a number of health-related topics (4), including monitoring epidemic spread of influenza and COVID-19 (5).

The analysis of user-generated information from social media has matured into a useful tool to monitor pandemic situations and understand public opinions during public health emergencies such as influenza and COVID-19. Several recent studies have analyzed influenza and COVID-19 related social media data from Twitter and Reddit using machine learning and deep learning methods for sentiment analyses to detect outbreaks and uncover themes of public concern (6–13). Specific studies focused on sentiment classification of influenza and COVID-19 tweets written in different languages, such as English (6,14), Arabic (15), and Nepali (16–18). In addition to sentiment analysis, an alternative method for monitoring influenza and COVID-19 trends on social media platforms is through the tracking of self-reports of symptoms and health experiences related to these diseases by users (19). This could offer useful complementary information for case detection. Previous studies have explored voluntary self-reporting of potential exposure to COVID-19 by Twitter users in the US and UK (20,21), but there is limited research on assessing user-level personal reports of influenza and COVID-19 in Canada. The first objective of our study is to assess whether English-speaking Canadian Twitter users disclose health experiences related to influenza and COVID-19 — such as potential exposure, symptoms, treatment and disease outcomes — that could be indicative of recent plausible influenza cases, ILIs or symptomatic COVID-19 infections. To support that process, we sought to construct ground-truth Twitter datasets for influenza and (separately) COVID-19 detection based on Twitter users’ reports in Saskatchewan, Canada.

Transformer-based pretrained language models (T-PTLMs) like Bidirectional Encoder Representations from Transformers (BERT) (22), RoBERTa (23) GPT-1 (24), and XLNet (25) have achieved great success in supporting Natural Language Processing (NLP) tasks (26). In recent years, T-PTLMs have further demonstrated outstanding performance on a number of NLP tasks in the public health domain (8,27,28). To enhance the performance of transformer-based language models in domain-specific or platform-specific tasks, various transformer-based language models have been pre-trained using either domain-specific or platform-specific data (26). BERTweet, BERTweet-covid, and COVID-Twitter BERT (CT-BERT) are notable social media-based T-PTLMs that were trained on Twitter data or Twitter data related to the COVID-19 pandemic. Despite the expectation that domain-specific language models would outperform general language models like BERT and RoBERTa in relevant NLP tasks, recent research findings in health-related social media text classification tasks have yielded mixed results. Some studies have shown that domain-specific language models do not necessarily outperform models pretrained on generic texts such as BERT or RoBERTa in sentiment analyses on COVID-19 tweets or general health-related text classification tasks (4,10). In this study, our second objective was to assess and compare the text classification performance of language models pretrained with either general knowledge text or domain-specific data on two social media-based text classification tasks. Specifically, we compared the performance of BERT and variants thereof — namely BERT, RoBERTa, BERTweet, and CT-BERT — in the context of influenza and COVID-19 infection detection using Twitter datasets. Additionally, due to class imbalances in our annotated Twitter datasets, we also explored the impact of sampling methods on the performance of these language models.

## 2. Materials and methods

### 2.1. Data collection, extraction and annotation

**Figure 1:**
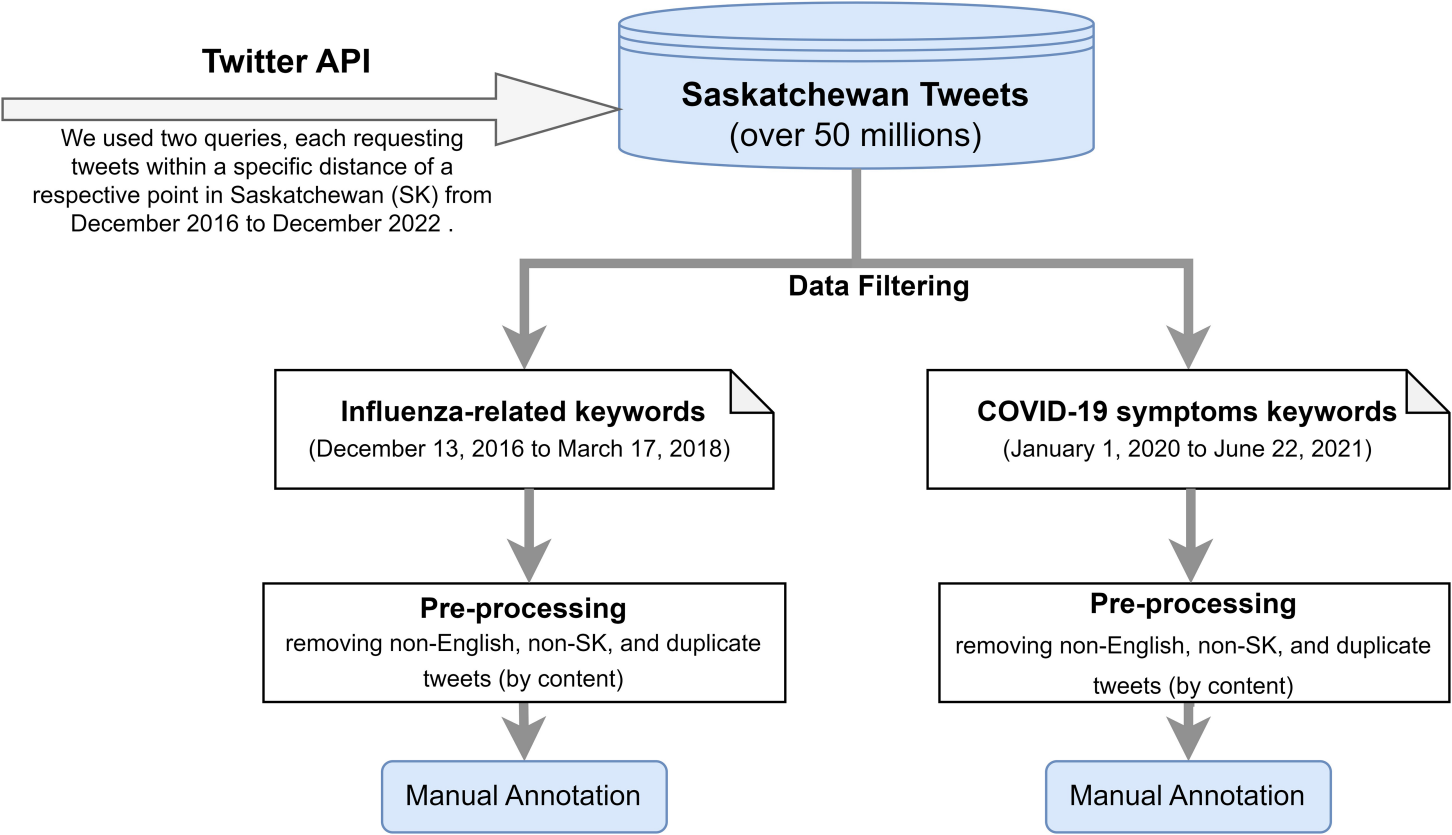
Overview of data collection and extraction processes.

We collected more than 50 million tweets using the Twitter application programming interface (API) between December 2016 and December 2022 in Saskatchewan (SK), Canada. We used two queries, each requesting tweets within a specific distance of a respective point in SK from December 2016 to December 2022. We then used a keyword-based filtering approach to extract relevant tweets that contained influenza-related keywords or COVID-19 symptom-related keywords. An overview of the data collection and extraction process is shown in Figure 1; we turn now to discuss the derivation of each dataset in turn.

The influenza-related Twitter dataset was extracted by filtering SK tweets using a set of influenza-related keywords from December 13, 2016 to March 17, 2018. The set of influenza-related keywords were developed based on surveillance standards for influenza and influenza-like illness (ILI), and can be broadly categorized as: (1) formal and informal names and agents of influenza and ILI (e.g., “swine flu”, “H1N1”, “H3N2”, “flu virus”, “influenza virus”, “Influenza B virus”, “seasonal influenza”, “cold”, etc.); (2) symptoms of influenza and flu-like illnesses (e.g., “fever”, “cough”, “sneeze”, “sore throat”, “runny or stuffy nose”, “muscle or body aches”, “muscle pain”, “myalgia”, “headaches”, “upper respiratory tract infection”, “acute respiratory illness”, “shortness of breath”, “malaise”, “fatigue”, “coryza”, etc); (3) flu complications (e.g., “vomiting”, “pneumonia”, “sinus infection”, “ear infection”); and (4) influenza prevention measures and treatments such as flu shots, antiviral drugs as designated using both their generic and brand names (e.g., “oseltamivir”, “zanamivir”, “peramivir”, “Tamiflu”).

The COVID-19 Twitter dataset was filtered using a set of COVID-19 symptom-related keywords from January 1, 2020 to June 22, 2021. We limited the keywords to COVID-19 symptoms only, as our objective was to identify tweets that contain information that are indicative of recent plausible symptomatic COVID-19 cases or COVID-19-like cases. The keyword list included “cough”, “shortness of breath”, “loss of taste and smell”, “difficulty of breathing”, “headache”, “chills”, “fever”, “fatigue”, “muscle aches”, “body ache”, “vomiting”, “nausea”, “throw up”, “diarrhea”, “abdominal pain”. We explored a variety of broader keywords associated with the COVID-19 pandemic, and we found that tweets filtered using terms specifically relating to COVID-19 symptoms were more relevant to the objective of our research. There are considerable similarities in symptoms between influenza and COVID-19; thus, when annotating the COVID-19 tweets, we consider plausible influenza cases or ILIs as “plausible COVID-19-like cases”.

Following the data cleansing process, 6,444 influenza-related tweets and 11,939 COVID-19 symptom-related tweets were retained for subsequent annotation. In this work, a team of 12 annotators systematically conducted manual annotation of pre-filtered tweets as to whether they described recent plausible influenza cases, ILIs or symptomatic COVID-19 infections. The annotation tasks were conducted independently for these two datasets. The influenza dataset was annotated by 8 annotators, whereas 7 annotators annotated the COVID-19 dataset. Three annotators contributed to the annotation tasks of both datasets. Table 1 provides a description of the respective annotation rules and guidelines for these two datasets to help the annotators distinguish the tweets. As the outcomes of this research is to support monitoring of users’ personal reports of health experiences associated with influenza and COVID-19, we did not regard news headlines, organizational tweets, or health promotion tweets as indicative of recent plausible influenza or COVID-19 cases. Tweet annotation required domain expertise; therefore, annotators were provided with a 1-hour training session covering the annotation task and key facts about influenza and COVID-19.

**Table 1:** Twitter datasets annotation tasks.

Annotation guidelines	Influenza twitter dataset	COVID-19 twitter dataset
Objective	Annotate tweets as to whether they suggest recent plausible influenza cases or influenza-like cases with one of the options below: 1. Definitely yes (recent convincing influenza cases or ILIs). 2. Maybe yes (recent plausible but not convincing influenza cases or ILIs). 3. Unsure. 4. Maybe no (unlikely recent plausible influenza cases or ILIs 5. Definitely no (not recent plausible influenza cases or ILIs) 6. I am not suitable to judge it, skip for now.	Annotate tweets as to whether they suggest recent plausible symptomatic COVID-19 or COVID-19-like (plausible influenza cases or ILIs) cases with one of the options below: 1. Definitely yes (recent convincing COVID-19 or COVID-19-like cases). 2. Maybe yes (recent plausible but not convincing COVID-19 or COVID-19-like cases). 3. Unsure. 4. Maybe no (unlikely recent plausible COVID-19 or COVID-19-like cases) 5. Definitely no (not recent plausible COVID-19 or COVID-19-like cases) 6. I am not suitable to judge it, skip for now.
Annotation Rules	1. Human subjects. It could implicate the user, someone the user might know, or people they encountered in public places. 2. The use of informal language and slang terms such as onomatopoeic expressions (e.g., “cough” or “cough cough”) should be scrutinized meticulously.	
	3. Flu-related advertisements, media news, health promotion-related tweets, general comments on influenza or flu shots won’t be considered as indicative of plausible influenza or influenza-like cases.	3. Organizational tweets, advertisements, media news, health promotion, general comments on COVID-19 and vaccine, and side effects after COVID-19 vaccine won’t be considered as indicative of plausible COVID-19 cases.
Tweet Examples	Examples of tweets inferring plausible influenza cases: “*Home with a cold flu surrounded by kleenex; blankets and some light reading material.*” “*Got a visit in last night with this sick little boy battling some flu like symptoms*”	Examples of tweets inferring plausible COVID-19 cases: “*Day 2 of self isolation - exhausted; fever is a bit worse but not out of control; painful body aches; cough is worsâ*” “*We have waited 4.5 hours for a callback from 811 after our youngest started throwing up and spiked a minor fever*”
Annotation Pre-Processing	None	The authors annotated tweets that they judged to clearly not suggest plausible COVID-19 cases based on the rules. 4398 tweets were considered not to suggest recent plausible COVID-19 cases. The remaining 7541 tweets were then annotated by volunteers.
Annotation Processes	Annotators underwent a comprehensive one-hour training session that covered the key facts about influenza or COVID-19 (including causes, symptoms, complications, drugs, vaccines) and guidelines for annotation. At the end of the session, the annotators took a quiz to assess their knowledge of the disease and understanding of the task.	

**Figure 2:**
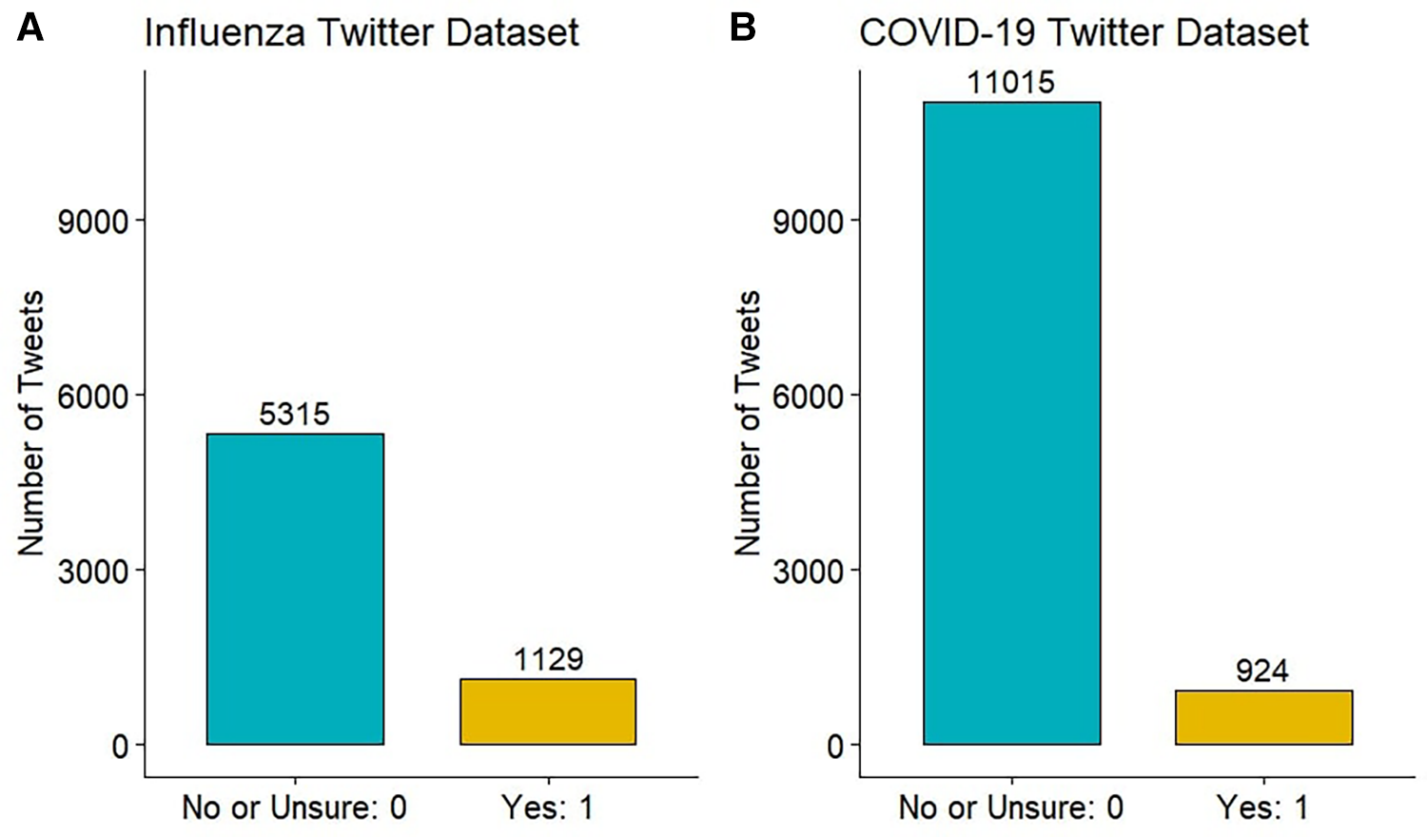
Distribution of annotations in Twitter datasets.

Each influenza-related tweet was annotated by three individuals, with at least one native English speaker among them. For those cases in which the majority was unable to establish a clear label, another annotator was brought in to re-annotate the tweet. The COVID-19 Twitter dataset was annotated slightly differently. One of the authors first evaluated all tweets, annotating those evidently not indicative of plausible COVID-19 or COVID-19-like cases as “No” (N=4,398). The remaining dataset (N=7,541) was assessed by two individuals, including at least one native English speaker. In the case of disagreement, the tweet was annotated by another annotator. For both datasets, majority voting was used to consolidate the tweet’s final binary annotation, which was either 1-“Yes” or 0-“No or Unsure”. Figure 2 shows the final distribution of annotations in the two datasets.

### 2.2. Data preprocessing

**Figure 3:**
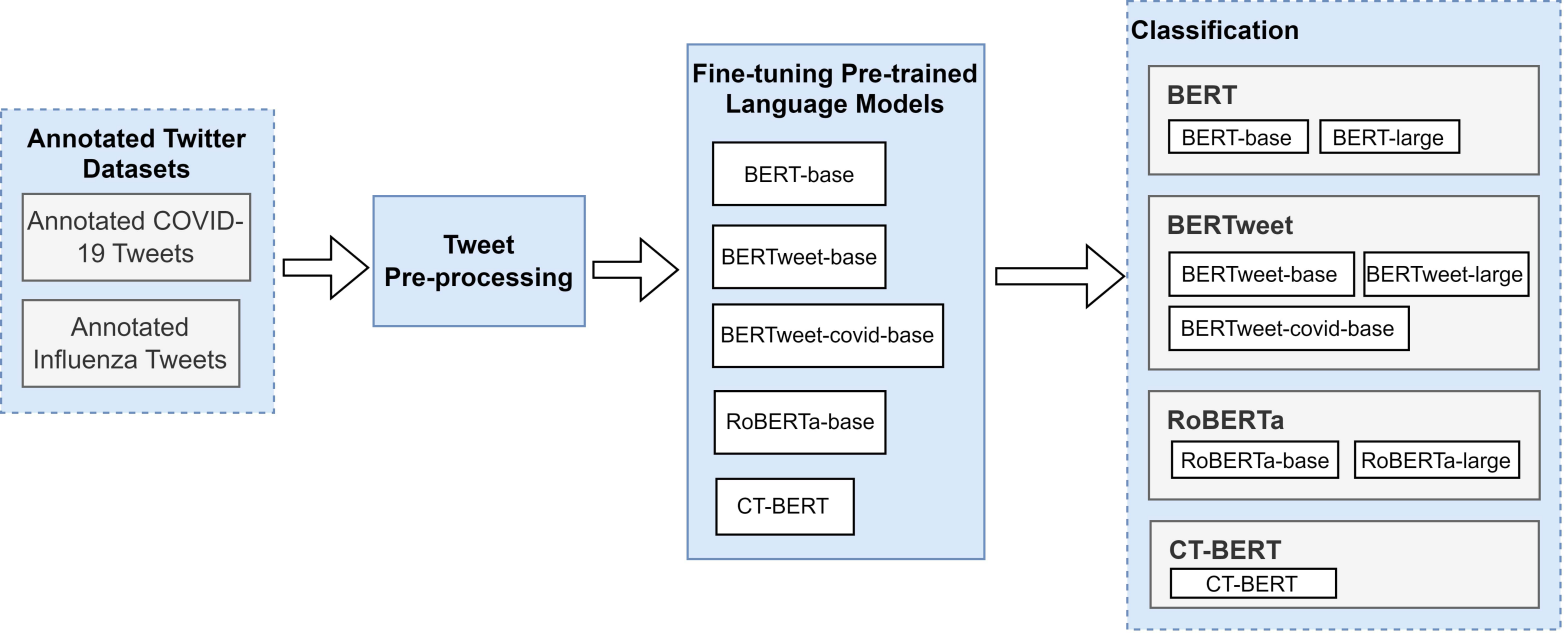
A high-level study workflow diagram.

Figure 3 presents the high-level study workflow diagram. Unlike formal text, tweets are characterized by their short and often unstructured nature. Without pre-processing, fine-tuning a pretrained language model with raw tweets may result in poor model performance (29). Therefore, we pre-processed the raw tweets with the following steps inspired by previous studies (30,31): (1) removal of emojis, (2) elimination of punctuation, links and mentions, (3) eliminating hashtags found at the end of tweets while retaining those in the middle after removing the “#” symbols, (4) removal of unnecessary spaces and newlines, (5) conversion to lowercase, (6) removal of empty tweets (resulting 40 tweets to be removed from COVID-19 dataset), and (7) loading the tokenizers from the pretrained models using the *Transformers* library (32). In our data-processing, we opted to exclude emojis, as we separately compared the performance of BERTweet and CT-BERT with and without emojis on the subset of tweets that contained emojis. With emojis being converted to their textual descriptions, we did not observe improvement in classification performance with emojis included.

Both datasets were split them into training and test subsets, respectively. We set 20% of each the datasets after data preprocessing as the test subset for that dataset. To address the class imbalance issue in both datasets, we applied oversampling and undersampling methods to each of the training subsets. Specifically, we used the *imbalanced-learn* Python library (33) to oversample the minority class and undersample the majority class, respectively. Table 2 presents the training and test sets for the influenza and COVID-19 Twitter datasets. We further set 10% of these two balanced training data subsets as validation data for training models.

**Table 2:** Training and test dataset sizes.

	Influenza twitter dataset		COVID-19 twitter dataset	
	“Yes” (Class 1)	“No or Unsure” (Class 0)	“Yes” (Class 1)	“No or Unsure” (Class 0)
Undersampled Training Set	899	899	755	755
Oversampled Training Set	4256	4256	8762	8762
Test Set	230	1059	169	2213

### 2.3. Transformer-based pretrained language models

Our study investigated the text classification performance of pre-trained BERT and three BERT variant models – RoBERTa, BERTweet, and CT-BERT using annotated influenza and COVID-19 Twitter datasets. Table 3 shows the eight BERT-based models employed in this study. We carried out our experiments using Google Colaboratory (34).

**Table 3:** BERT and BERT variant models used in the study.

Model	Architecture	Task	Model versions	Pretraining corpus-based	Pretraining data	Total parameters
BERT	Multi-layer bidirectional transformer encoder	MLM, NSP	BERT-base, BERT-large	general-domain	800 M words (BooksCorpus) and 2500 M words (English Wikipedia), 16GB of uncompressed text.	base: 110 M; large: 340M
RoBERTa	BERT with improved pretraining procedures	MLM	RoBERTa-base, RoBERTa-large	general-domain	data used to train BERT, 63 M English news articles (CC-NEWS), OpenWebText, and Stories; 160GB of uncompressed text.	base: 124 M; large: 355 M
BERTweet	BERT-base model with RoBERTa’s pre-training procedure.	MLM	BERTweet-base, BERTweet-large, BERTweet-covid-base	social media-based, domain-specific	BERTweet-base and large: 845M English Tweets and 5 M COVID-19 tweets; BERTweet-covid-base: 23 M COVID-19 English tweets.	base: 135 M; large: 355 M
COVID-Twitter-BERT	BERT-large	MLM, NSP	COVID-Twitter-BERT-v2	social media-based, domain-specific	160 M COVID-19 English tweets	340 M

**MLM**: Masked Language Modeling; **NSP**: Next Sentence Prediction

**BERT** Bidirectional Encoder Representations from Transformers (BERT) is a language representation model using the widely adopted transformer architecture (35). Given the wide use of transformers, we will omit an exhaustive description of its architecture and direct readers to Vaswani et al. (35) on the details. BERT is pre-trained on two unsupervised tasks: Masked Language Modeling (MLM) and Next Sentence Prediction (NSP) (22). MLM is an unsupervised task where certain tokens in a text sequence are randomly masked, and the model aims to predict these masked segments of the text. NSP is a task that the model predicts whether a given pair of sentences in a text document are consecutive. The pre-training corpus of BERT includes BooksCorpus and Wikipedia datasets (16GB). BERT has many variants models with different model sizes. We used BERT-base and BERT-large in this study with parameters of 110M and 340M, respectively.

**RoBERTa** Robustly Optimized BERT Pretraining Approach (RoBERTa) (23) is an extension of BERT and has the same architecture as BERT. RoBERTa can be seen as an enhanced version of BERT with improved pretraining procedures and additional pre-training data. RoBERTa applies a dynamic masking approach and removes the NSP objective, which sets it apart of BERT. RoBERTa’s training time is longer and utilizes a larger corpora in comparison to BERT. RoBERTa is pretrained on a total of 160GB of text data, including the original data used to train BERT (BooksCorpus and Wikipedia), CC-News, OpenWebText, and Stories. For our experiments on COVID-19 and influenza Twitter datasets, we used RoBERTa-base and RoBERTa-large models, which include 124M and 355M parameters, respectively.

**BERTweet** BERTweet (36) is a variant of BERT trained with a masked language modeling objective. Using the same architecture as BERT-base, BERTweet is trained to process text data from Twitter. The pre-training procedure of BERTweet is based on RoBERTa for improved performance. After preprocessing the raw tweets, BERTweet used 845M English tweets and 5M English tweets related to the COVID-19 pandemic (from 01/2020 to 03/2020) to train BERTweet-base and BERTweet-large models. The total pretraining data used for these models amounts to approximately 80GB (16B word tokens). Subsequently, a corpus of 23M COVID-19 related English tweets was collected, and the authors continued pre-training using the base version of pre-trained BERTweet (36). This led to the development of the BERTweet-covid-base model. This study used the pre-trained BERTweet-base, BERTweet-covid-base (uncased), and BERTweet-large, which have 135M, 135M, and 355M parameters, respectively.

**CT-BERT** COVID-Twitter-BERT (CT-BERT) (37) is a transformer-based model that utilizes the BERT-large architecture. It underwent pretraining on a corpus of 160M English tweets specifically focused on COVID-19, covering the period from January 12 to April 16, 2020. The first version of CT-BERT is pretrained on a corpus of 160M English tweets related to COVID-19 from January 12 to April 16, 2020. Before the pretraining process, the original tweets were pre-processed, which involved cleaning the tags, replacing the username and URLs, and eliminating duplicate tweets. The final corpus contains 22.5M tweets (0.6 billion words). The second version of the CT-BERT is identical to the first version except that it was trained on more COVID-19 English tweets collected until July 5, 2020. In this study, we used the CT-BERT v2 version, which was pretrained on a larger COVID-19 Twitter dataset.

### 2.4. Fine-tuning

Fine-tuning T-PTLMs involves training the pretrained models on a specific downstream task. This is done by adding a dense layer and a task-specific output layer for adjusting model parameters on a task-specific dataset. The models are initialized with pretrained parameters and then fine-tune the entire neural network by using gradient descent-based backpropagation technology to find the optimal hyperparameter values (38).

In our study, we fine-tuned BERT-base, RoBERTa-base, BERTweet-base, BERTweet-covid-base, and CT-BERT. Inspired by previous studies (22,23), we experimented with learning rates of {5e−5,3e−5,2e−5,1e−5}, batch sizes of {16,32}, and epochs of {2,3,4} with a basic grid search strategy. For BERT-large, RoBERTa-large, and BERTweet-large, we used the same hyperparameter values as in their respective base versions. Finally, the Adam optimizer (39) was employed for all models, and we measured the categorical cross-entropy and categorical accuracy during fine-turning. The optimal hyperparameter values for each model are presented in Table 4.

**Table 4:** Optimal parameters used for BERT and BERT variant models.

	BERT		RoBERTa		BERTweet		BERTweet-covid		CT-BERT	
	Covid	Flu	Covid	Flu	Covid	Flu	Covid	Flu	Covid	Flu
Learning Rate	1e−5	3e−5	1e−5	3e−5	3e−5	2e−5	1e−5	3e−5	1e−5	3e−5
Epochs	4	3	4	4	4	4	3	3	4	3
Batch Size	32	16	32	16	32	16	32	16	32	16

Covid represents the COVID-19 Twitter dataset; Flu represents the influenza Twitter dataset

### 2.5. Evaluation metrics

Model performance was evaluated using recall, F1 score, accuracy, and area under the receiver operating characteristic curve (AUC). To obtain a more reliable evaluation of the models’ performance, we conducted five separate runs with varying initialization for each model and dataset, while maintaining invariant the values of the fine-tuning hyperparameters. This is due to the instability of the fine-tuning process, as the classification performance can vary significantly when training the same model with different random seeds (40). The resulting classification performance metrics (recall, F1, accuracy, and AUC) were averaged over the test subsets of the COVID-19 and influenza Twitter datasets, respectively. Additionally, we performed independent one-sided T-tests to compare the model performance for each sampling method.

## 3. Results and analysis

### 3.1. Exploratory data analysis

**Figure 4:**
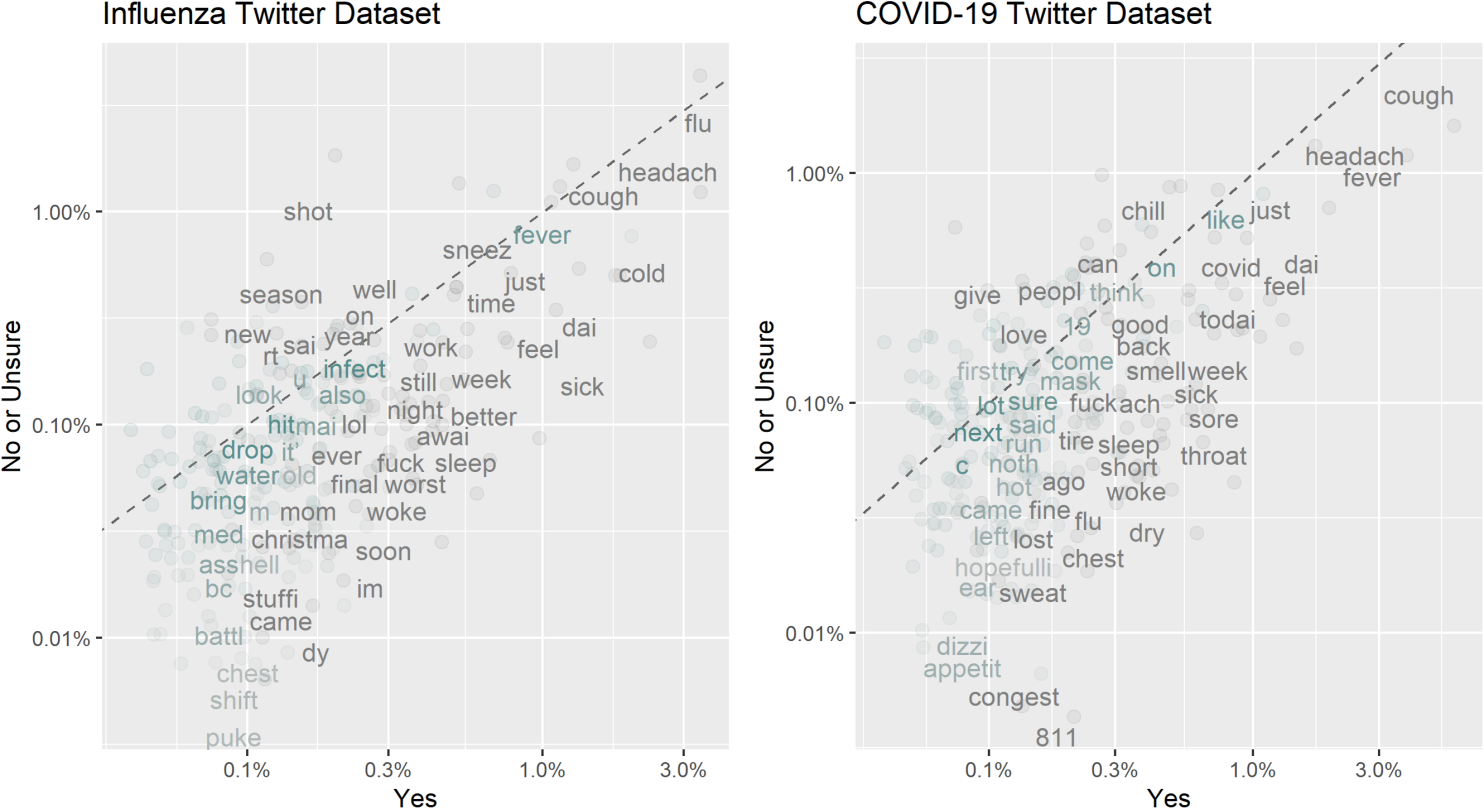
Frequencies of the top 250 word stems by different annotations.

We constructed two datasets of annotated tweets based on users’ reports of recent plausible influenza or COVID-19 infections. Each tweet was annotated by no fewer than two annotators, with the final annotation determined through the majority rule. Of the 6444 influenza-related tweets, 1129 (17.5%) were annotated as “Yes” — suggesting recent plausible influenza cases or ILIs. Of the 11939 COVID-19 symptom-related tweets, 924 (7.7%) were annotated as “Yes” — suggesting recent plausible symptomatic COVID-19 or COVID-19-like cases. Figure 4 illustrates the frequencies of the top 250 word stems in the influenza and COVID-19 datasets. Word stems located far from the dotted line were more prevalent in one class than the other, while those that were close to the line had similar frequencies in both the “Yes” and “No or Unsure” classes. The word stems were extracted by removing links and usernames, tokenizing the tweets, stemming the words and removing stop words. In Figure 4, we observe that several symptom-related word stems (such as “sick”, “puke”, “fever”, “sore”, “cough”) exhibit higher frequencies in the “Yes” class. In the COVID-19 Twitter dataset, the number “811” having a higher frequency in the “Yes” class represents Saskatchewan’s HealthLine number, which was widely promoted as a source of non-urgent medical advice and health information for COVID-19.

### 3.2. Comparison of model performance

The performance of the models under various sampling methods is presented in Tables 5 and 6. The highest-ranked value of each metric across the four models given a specific sampling method and model size group (“base” or “large”) was marked as the reference group (“ref”) for both datasets. To assess the model performance, independent one-sided t-tests were conducted to compare the results of each model to the highest ranked one (the reference group) for the same metric, sampling method and dataset across language models in a given model size group (“base” or “large”). Additionally, for each Twitter dataset, we compared the performance of different sampling methods for each model, as well as the performance of eight models within each sampling method regardless of the model size group. These comparisons can be found in Tables S1 and S2 in the Supplementary Materials.

**Table 5:** Performance metrics of models on COVID-19 Twitter datasets for each sampling method.

Size	Model	Mean Recall			Mean F1			Mean AUC			Mean Accuracy		
		N	O	U	N	O	U	N	O	U	N	O	U
Base	BERT-base	92.6	91.4${}^{*}$	74${}^{*}$	91.8${}^{*}$	92${}^{*}$	80.2${}^{*}$	63${}^{*}$	74.1	70${}^{*}$	92.7${}^{*}$	91.6${}^{*}$	74.2${}^{*}$
	BERTweet-base	92.8	93	82.6	93	**93.4(ref)**	86.6	76.7	78	**87(ref)**	92.9	**93.1(ref)**	82.6
	BERTweet-covid-base	**93.4(ref)**	**93.2(ref)**	**85.2(ref)**	**93.6(ref)**	93.4	**88.2(ref)**	79.5	**80.5(ref)**	86.5	**93.6(ref)**	93.1	**85.3(ref)**
	RoBERTa-base	92.8	92.6	85.2	93.2	92.8${}^{*}$	88	**80(ref)**	80.2	84.7${}^{*}$	92.8	92.6	85.1
Large	BERT-large	94${}^{*}$	93.2${}^{*}$	81.2	93.6${}^{*}$	93.4${}^{*}$	85.4	72	77.6${}^{*}$	82.6${}^{*}$	93.9	93.3	81.3
	BERTweet-large	93.2${}^{*}$	91.2${}^{*}$	82.6	93.2${}^{*}$	91.2${}^{*}$	86	73	**82.8(ref)**	84.6	93.2${}^{*}$	91.3${}^{*}$	82.4
	CT-BERT	**94.8(ref)**	**94.2(ref)**	**85.2(ref)**	**94.4(ref)**	**94.2(ref)**	**88(ref)**	**77.2(ref)**	81	**86.2(ref)**	**94.6(ref)**	**94(ref)**	**85.2(ref)**
	RoBERTa-large	94${}^{*}$	93${}^{*}$	85	94.2	93.6${}^{*}$	88	76.9	81.3	85.8	94.1	93.1${}^{*}$	85

The bold values just highlight the “ref” group. **ref**: the reference group used for the t-test, and it is also the highest ranked value given a sampling method for a specific metric across different language models in either the “base” or “large” model size; ${}^{*}$: the metric is statistically different from the reference group for a given sampling method and metric across language models in the “base” or “large” model size; **N**: no sampling; **O**: random oversampling; **U**: random undersampling.

**Table 6:** Performance metrics of models on Influenza Twitter datasets for each sampling method.

Size	Model	Mean Recall			Mean F1			Mean AUC			Mean Accuracy		
		N	O	U	N	O	U	N	O	U	N	O	U
Base	BERT-base	84.2${}^{*}$	85.2${}^{*}$	76.6${}^{*}$	83${}^{*}$	85.4${}^{*}$	79.2${}^{*}$	68.2${}^{*}$	75.3${}^{*}$	78.9${}^{*}$	84.3${}^{*}$	85.1${}^{*}$	76.5${}^{*}$
	BERTweet-base	90.8	**91.8(ref)**	87.4	91	**92(ref)**	88.2	**87.1(ref)**	**88.6(ref)**	88.9	90.9	**91.9(ref)**	87.4
	BERTweet-covid-base	**91.6(ref)**	91.6	87.6	**91.6(ref)**	91.6	88.4	84.9	86.6	**89(ref)**	**91.6(ref)**	91.7	87.5
	RoBERTa-base	90.6${}^{*}$	90.4${}^{*}$	**88(ref)**	90.8${}^{*}$	90.8${}^{*}$	**88.6(ref)**	86.3	85.9${}^{*}$	88.3	90.6${}^{*}$	90.5${}^{*}$	**87.8(ref)**
Large	BERT-large	89.8${}^{*}$	89.6${}^{*}$	82.6${}^{*}$	89.4${}^{*}$	89.6${}^{*}$	84${}^{*}$	81.3${}^{*}$	81.7${}^{*}$	84.8${}^{*}$	89.7${}^{*}$	89.8${}^{*}$	82.6${}^{*}$
	BERTweet-large	**92.4(ref)**	**92.2(ref)**	85.4	**92.6(ref)**	**92.2(ref)**	86.6	**87.6(ref)**	87.1	**88(ref)**	**92.4(ref)**	**92.2(ref)**	85.5
	CT-BERT	90.6${}^{*}$	91.2	**87.4(ref)**	90.4${}^{*}$	91.4	**88(ref)**	85.3	**88.1(ref)**	86.5	90.5${}^{*}$	91.1	**87.4(ref)**
	RoBERTa-large	90.8${}^{*}$	89.2${}^{*}$	83.8${}^{*}$	90.6${}^{*}$	89.8${}^{*}$	85.4${}^{*}$	85.1	87.6	87.4	90.7${}^{*}$	89.2${}^{*}$	83.9${}^{*}$

The bold values just highlight the “ref” group. **ref** the reference group used for the t-test, and it is also the highest ranked value given a sampling method for a specific metric across different language models in either the “base” or “large” model size; ${}^{*}$: the metric is statistically different from the reference group for a given sampling method and metric across language models in the “base” or “large” model size; **N**: no sampling; **O**: random oversampling; **U**: random undersampling.

#### 3.2.1. Performance metrics evaluation of COVID-19 tweet classification

Model performance on the COVID-19 Twitter dataset is presented in Table 5. Among the four models in the “base” model size group, the BERTweet-covid-base model consistently achieved the highest or equally highest mean scores in recall, F1, and accuracy across the various sampling methods. The performance of the BERTweet-base and RoBERTa-base models was similar to that of the BERTweet-covid-base model with no statistically significant difference. In contrast, the BERT-base model exhibited statistically worse performance in mean F1 and mean accuracy compared to the BERTweet-covid-base model.

Among the models in the “large” size group, the CT-BERT model achieved the highest mean scores in recall, F1, and accuracy across all sampling methods. However, there were no statistically differences in mean scores of recall, F1 and accuracy among the large models when the undersampling method was used. The CT-BERT model demonstrated statistically significant better performance in mean recall and F1 where either the oversampling method or no sampling was applied. Notably, the CT-BERT model showed significant improvement in mean recall and F1 compared to its base model, BERT-large.

#### 3.2.2. Performance metrics evaluation of influenza tweet classification

Table 6 presents the model performance on the Influenza Twitter dataset. When undersampling method was employed, RoBERTa-base had the highest mean scores in recall (88%), F1 (88.6%), and accuracy (87.8%) in the “base” model size group. BERTweet-base, BERTweet-covid-base had comparable performance to the RoBERTa-base model with no statistically significant difference. When no sampling or oversampling was applied, both the BERTweet-base model and BERTweet-covid-base model had better and comparable performance in mean recall, F1, and accuracy measures with no statistical difference observed between them. The RoBERTa-base and BERT-base models exhibited significantly inferior performance in the “base” model size group with no sampling or oversampling method.

In the “large” model size group, BERTweet-large model emerged as the top performer under no sampling or oversampling method, delivering the best mean scores in recall, F1, and accuracy. While the BERT-large model consistently underperformed in the “large” model size group regardless of the sampling method used.

#### 3.2.3. Base models, large models, and sampling approach

In this study, we used the base and large models of BERT, RoBERTa, and BERTweet. The key similarity between the base and large versions of a pre-trained model lies in their identical architecture and pretraining data. The main difference between the base and large models is their size and computational requirements. All three pretrained base models include 12 transformer layers with over 100 million parameters. By contrast, the large version of the pretrained models include double transformer layers than the base version with over 300 million parameters. Therefore, large models need larger computer resources.

Upon comparing the performance of BERT-base and BERT-large models on the COVID-19 and influenza Twitter datasets, we found that the BERT-large model outperformed BERT-base in all four evaluation metrics. However, the performance of the RoBERTa and BERTweet models — whether in their base or large forms — varied depending on the datasets and the sampling approach.

With respect to the sampling approach, the results in Table S2 (found in the Supplementary Materials) suggest that, when compared to the undersampling method, the classification performance (as measured by mean recall, F1, and accuracy scores) for both the COVID-19 and influenza Twitter datasets was generally better with no sampling or oversampling method. The comparison between no sampling and the oversampling approach revealed varied results across models and datasets. The oversampling method did not significantly enhance model performance on the COVID-19 Twitter dataset, and in certain cases, it even led to poorer performance, as observed with the RoBERTa-large model. On the other hand, for the influenza Twitter dataset, there was no statistically significant difference in mean recall, F1, or accuracy between the oversampling and no sampling methods across the models. In addition, it is worth noting that for the mean AUC scores, the undersampling method yielded better performance for several models.

## 4. Discussion

This study investigated whether Twitter users disclose health-related experiences that may suggest recent plausible cases of influenza and COVID-19 in Saskatchewan, Canada. We collected and manually annotated pre-filtered tweets for influenza and COVID-19 detection, and applied pre-trained BERT and BERT variant models to classify the tweets. We achieved an F1 score of up to 94.4% on the COVID-19 Twitter dataset and 92.6% on the influenza Twitter dataset. To the best of our knowledge, our study is the first to examine the Twitter users’ reports of health experiences that could be indicative of influenza and COVID-19 infections in Canada. Previous studies have assessed Twitter users’ self-reports of plausible COVID-19 exposure or infections for tracking COVID-19 spread in the US and UK (20,21), and other Canadian studies using COVID-19 related Twitter data are focused on analyzing organizational tweets (41), COVID-19 tweets posted by federal members of parliament (42), or more general sentiment analysis using Twitter emojis (43).

In this study, we evaluated the performance of per-trained BERT and three BERT-variant language models (RoBERTa, BERTweet, and CT-BERT) on our annotated influenza and COVID-19 Twitter datasets, respectively. To address the issue of imbalanced data in classification, we explored data-level sampling approaches to balance the imbalanced class distribution, which include oversampling to augment data in the minority classes and undersampling to decrease data in the majority classes. Our analyses showed that undersampling the training datasets for both classification tasks resulted in significantly worse model performance in mean recall, F1 score, accuracy, while the impact on AUC scores varied by dataset. When the oversampling approach was used, there was no substantial improvement in the mean F1 scores across the models in comparison to the results obtained with no sampling on both datasets. We also found that domain-specific models performed better than general language models on our Twitter datasets. The CT-BERT model, trained on COVID-19 related tweets, demonstrated superior performance on the COVID-19 Twitter dataset with both oversampling and no sampling methods. However, in the context of predicting plausible influenza cases, the CT-BERT model did not consistently outperform the BERTweet-large model, despite the similarities in symptoms between COVID-19 and influenza. This aligns with previous findings that CT-BERT outperforms other models in COVID-19 related classification tasks (37). It is worth noting that when assessing the performance of CT-BERT on non-COVID-19 related Twitter data – the influenza Twitter dataset – its performance is comparable or slightly inferior to the BERTweet-large model, which was pre-trained on general Twitter language rather than exclusively COVID-19 related language. Furthermore, the BERT models performed worse than domain-specific models on both classification tasks.

We further compared the performance of the base and large versions of the BERT, RoBERTa, and BERTweet models. Despite the far larger parameter count of large models, we did not observe a consistent trend of large models outperforming their base counterparts. Although BERT-large demonstrated better performance than BERT-base, RoBERTa-base and BERTweet-base models yielded mixing results to their large counterparts on our Twitter datasets across different sampling method. One possible reason for this could be that the datasets used for fine-tuning are insufficiently large to fully utilize the capacity of the large models. As a result, the large models may end up overfitting to the training data, precipitating little performance improvement on the test sets. Considering that large models require more computational resources and longer training time, using the base models may suffice for many practical applications.

This study is subject to several limitations. Firstly, the size of our Twitter datasets was relatively small, and we restricted our analysis to English-language tweets from Saskatchewan, Canada. Therefore, our findings may not be generalizable to other jurisdictions. Secondly, we only used symptom-related keywords to filter COVID-19 tweets, instead of using more comprehensive keywords related to the COVID-19 pandemic. This was done to render more manageable the number of tweets requiring manual annotation, but may have overlooked other types of self-reporting related to COVID-19 infections that are not based on symptoms, or that use other terms for symptoms. In addition, although monitoring of individuals’ self-reporting of symptoms and health experiences related to influenza and COVID-19 through social media presents an opportunity to complement real-time disease surveillance, it has its own limitations. One such limitation is the inability to detect asymptomatic cases of COVID-19, which is estimated to represent 40.5% of the population with confirmed COVID-19 infections (44). Recent changes to the size and character of the twitterverse and the rapid escalation in cost for production-scale broader tweet monitoring also raise concerns. Furthermore, since both the influenza and COVID-19 datasets exhibited a high frequency of symptom-related words, monitoring the disease spread through this method may not be sufficiently timely given the incubation periods of both infections, and merits combination with less highly lagged surveillance data sources, such as those involve wastewater assays (45).

Despite such limitations, the classification methods presented here merit consideration for complementing existing sentinel and syndromic surveillance methods, for which the high temporal velocity of tweets and low reporting delays offer particular attractions. Of particular note are the potential for such classification methods to produce time series to inform health system modeling consuming high-velocity data, such as achieved strong use during the COVID-19 pandemic for situational awareness and short-term forecasting in Saskatchewan and for each Canadian province and Canadian Reserves (46).

## 5. Conclusion

Leveraging domain-specific language models to monitor users’ reports of health experiences related to influenza and COVID-19 infections on social media offers the potential to supplement real-time disease surveillance. The BERT family of pretrained language models offer competitive classification performance across multiple measures, with CT-BERT supporting modestly higher performance for COVID-related tweets than other language models, but performing in an undistinguished manner when classifying influenza-related tweets. Despite a number of limitations, such methods demonstrate sufficient promise to merit consideration as one element of the broader communicable disease surveillance toolbox increasingly important for adaptive disease prevention and control in the future.

## Data Availability

The Twitter datasets used in the study were publicly available at the time of data collection. This data can be found here: https://github.com/tianyuan09/InfluenzaCOVID-19TwitterDatasets.
