# Supplementary material for: Comparison of pretrained transformer-based models for influenza and COVID-19 detection using social media text data in Saskatchewan, Canada
Source: Front Digit Health. 2023 Jun 28;5:1203874. doi: 10.3389/fdgth.2023.1203874 (PMC10338115; doi:10.3389/fdgth.2023.1203874)
Supplement: Supplementary file 1 [file Table1.pdf]

Supplementary Material

1 SUPPLEMENTARY TABLES

Table S1. Comparison of model performance metrics within each sampling method on COVID-19 and influenza Twitter datasets

Sampling	Size	Model	COVID-19 Twitter Dataset				Influenza Twitter Dataset			
			Mean Recall	Mean F1	Mean AUC	Mean Accuracy	Mean Recall	Mean F1	Mean AUC	Mean Accuracy
None	Base	BERT-base	92.6*	91.8*	63*	92.7*	84.2*	83*	68.2*	84.3*
		BERTweet-base	92.8*	93*	76.7	92.9*	90.8	91*	87.1	90.9
		BERTweet-covid-base	93.4*	93.6	79.5	93.6*	91.6*	91.6*	84.9	91.6*
		RoBERTa-base	92.8*	93.2*	**80(ref)**	92.8*	90.6*	90.8*	86.3	90.6*
	Large	BERT-large	94*	93.6*	72*	93.9	89.8*	89.4*	81.3*	89.7*
		BERTweet-large	93.2*	93.2*	73	93.2*	**92.4(ref)**	**92.6(ref)**	**87.6(ref)**	**92.4(ref)**
		CT-BERT	**94.8(ref)**	**94.4(ref)**	77.2	**94.6(ref)**	90.6*	90.4*	85.3	90.5*
		RoBERTa-large	94*	94.2	76.9	94.1	90.8*	90.6*	85.1	90.7*
Oversampling	Base	BERT-base	91.4*	92*	74.1*	91.6*	85.2*	85.4*	75.3*	85.1*
		BERTweet-base	93*	93.4*	78*	93.1*	91.8	92	**88.6(ref)**	91.9
		BERTweet-covid-base	93.2*	93.4*	80.5	93.1*	91.6	91.6	86.6	91.7
		RoBERTa-base	92.6*	92.8*	80.2	92.6*	90.4*	90.8*	85.9*	90.5*
	Large	BERT-large	93.2*	93.4*	77.6*	93.3	89.6*	89.6*	81.7*	89.8*
		BERTweet-large	91.2*	91.2*	**82.8(ref)**	91.3*	**92.2(ref)**	**92.2(ref)**	87.1	**92.2(ref)**
		CT-BERT	**94.2(ref)**	**94.2(ref)**	81	**94(ref)**	91.2	91.4	88.1	91.1
		RoBERTa-large	93*	93.6*	81.3	93.1*	89.2*	89.8*	87.6	89.2*
Undersampling	Base	BERT-base	74*	80.2*	70*	74.2*	76.6*	79.2*	78.9*	76.5*
		BERTweet-base	82.6	86.6	**87(ref)**	82.6	87.4	88.2	88.9	87.4
		BERTweet-covid-base	**85.2(ref)**	**88.2(ref)**	86.5	**85.3(ref)**	87.6	88.4	**89(ref)**	87.5
		RoBERTa-base	85.2	88	84.7*	85.1	**88(ref)**	**88.6(ref)**	88.3	**87.8(ref)**
	Large	BERT-large	81.2*	85.4*	82.6*	81.3*	82.6*	84*	84.8*	82.6*
		BERTweet-large	82.6	86	84.6	82.4	85.4	86.6	88*	85.5
		CT-BERT	85.2	88	86.2	85.2	87.4	88	86.5	87.4
		RoBERTa-large	85	88	85.8	85	83.8*	85.4*	87.4*	83.9*

ref: the reference group used for the t-test, and it is also the highest ranked value for the same dataset, sampling method, and metric across language models; *: the metric is statistically different from that of the reference group for the same dataset and sampling method across language models.

Table S2. Comparison of model performance metrics for different sampling methods on COVID-19 and influenza Twitter datasets

Model	Sampling	COVID-19 Twitter Dataset				Influenza Twitter Dataset			
		Mean Recall	Mean F1	Mean AUC	Mean Accuracy	Mean Recall	Mean F1	Mean AUC	Mean Accuracy
BERT-base	N	**92.6(ref)**	91.8	63*	**92.7(ref)**	84.2	83	68.2*	84.3
	O	91.4*	**92(ref)**	**74.1(ref)**	91.6*	**85.2(ref)**	**85.4(ref)**	75.3	**85.1(ref)**
	U	74*	80.2*	70	74.2*	76.6*	79.2*	**78.9(ref)**	76.5*
BERTweet-base	N	92.8	93	76.7*	92.9	90.8	91	87.1*	90.9
	O	**93(ref)**	**93.4(ref)**	78*	**93.1(ref)**	**91.8(ref)**	**92(ref)**	88.6	**91.9(ref)**
	U	82.6*	86.6*	**87(ref)**	82.6*	87.4*	88.2*	**88.9(ref)**	87.4*
BERTweet-covid-base	N	**93.4(ref)**	**93.6(ref)**	79.5*	**93.6(ref)**	**91.6(ref)**	**91.6(ref)**	84.9*	91.6
	O	93.2	93.4	80.5*	93.1	91.6	91.6	86.6*	**91.7(ref)**
	U	85.2*	88.2*	**86.5(ref)**	85.3*	87.6*	88.4*	**89(ref)**	87.5*
RoBERTa-base	N	**92.8(ref)**	**93.2(ref)**	80*	**92.8(ref)**	**90.6(ref)**	**90.8(ref)**	86.3	**90.6(ref)**
	O	92.6	92.8	80.2*	92.6	90.4	90.8	85.9*	90.5
	U	85.2*	88*	**84.7(ref)**	85.1*	88*	88.6*	**88.3(ref)**	87.8*
BERT-large	N	**94(ref)**	**93.6(ref)**	72*	**93.9(ref)**	**89.8(ref)**	89.4	81.3	89.7
	O	93.2	93.4	77.6*	93.3	89.6	**89.6(ref)**	81.7*	**89.8(ref)**
	U	81.2*	85.4*	**82.6(ref)**	81.3*	82.6*	84*	**84.8(ref)**	82.6*
BERTweet-large	N	**93.2(ref)**	**93.2(ref)**	73*	**93.2(ref)**	**92.4(ref)**	**92.6(ref)**	87.6	**92.4(ref)**
	O	91.2*	91.2*	82.8	91.3*	92.2	92.2	87.1	92.2
	U	82.6	86	**84.6(ref)**	82.4	85.4*	86.6*	**88(ref)**	85.5*
CT-BERT	N	**94.8(ref)**	**94.4(ref)**	77.2*	**94.6(ref)**	90.6	90.4	85.3	90.5
	O	94.2*	94.2	81*	94*	**91.2(ref)**	**91.4(ref)**	**88.1(ref)**	**91.1(ref)**
	U	85.2*	88*	**86.2(ref)**	85.2*	87.4*	88*	86.5	87.4*
RoBERTa-large	N	**94(ref)**	**94.2(ref)**	76.9*	**94.1(ref)**	**90.8(ref)**	**90.6(ref)**	85.1	**90.7(ref)**
	O	93*	93.6*	81.3*	93.1*	89.2	89.8	**87.6(ref)**	89.2
	U	85*	88*	**85.8(ref)**	85*	83.8*	85.4*	87.4	83.9*

ref: the reference group used for the t-test, and it is also the highest ranked value for the same dataset, model and metric across different sampling methods; *****: the metric is statistically different from that of the reference group for the same dataset and model; **N**: no sampling; **O**: random oversampling; **U**: random undersampling.
